# Pathological Features and Genotyping of *Mycobacterium avium sub* spp. *paratuberculosis* (MAP) in Small Ruminants in Saudi Arabia

**DOI:** 10.3390/pathogens15040355

**Published:** 2026-03-27

**Authors:** Hassan Albaqshi, Mahmoud Hamouda, Yahya Aljasem, Reham Karam, Fahad A. Al Hizab

**Affiliations:** 1 Department of Pathology, College of Veterinary Medicine, King Faisal University, Al-Ahsa 31982, Saudi Arabia; 2 Weqaa Center, Central Veterinary Laboratory, Riyadh 11454, Saudi Arabia; 3 Virology Department, Faculty of Veterinary Medicine, Mansoura University, Mansoura 35516, Egypt

**Keywords:** IS900 real-time PCR, serological detection, molecular typing, subclinical infection, small ruminant herds

## Abstract

Johne’s disease, caused by *Mycobacterium avium* subsp. paratuberculosis (MAP), is endemic in Saudi Arabia and contributes to substantial production losses in small-ruminant herds. This study investigated MAP infection in 388 locally raised small ruminants (151 sheep and 237 goats) using IS900 real-time PCR (qPCR) on fecal samples and indirect ELISA on serum samples. Ziehl–Neelsen (ZN) staining and pathological assessment were applied as supportive tools in necropsied cases. Overall, qPCR detected MAP DNA in 135/388 animals (34.8%), with higher positivity in goats (100/237; 42.1%) than in sheep (35/151; 23.1%). ELISA detected MAP antibodies in 120/388 animals (30.9%), including 90/237 goats (37.9%) and 30/151 sheep (19.8%). Based on clinical examination (presence/absence of clinical signs), qPCR-positive animals were categorized as subclinical (n = 15; 10 goats and 5 sheep) or clinically progressed (n = 120; 90 goats and 30 sheep). Gross and histopathological findings were assessed in a necropsied subset (n = 20; 10 subclinical and 10 clinically progressed), revealing mild focal granulomatous enteritis with scant acid-fast bacilli in subclinical cases and diffuse lepromatous-type granulomatous lesions with abundant bacilli in clinically progressed animals. Genotyping and subtyping were performed on tissue-derived DNA from six necropsied cases using DMC, IS900, and F57 targets, and IS900 sequencing confirmed the circulation of both Type C/II and Type S/I MAP strains. Collectively, these findings demonstrate widespread MAP infection among small ruminants in Saudi Arabia, with higher detection rates and more pronounced pathology in goats, and highlight the genetic heterogeneity of circulating MAP strains.

## 1. Introduction

Johne’s disease, or paratuberculosis, is a progressive wasting illness caused by *Mycobacterium avium* subspecies *paratuberculosis* (MAP), a slow-growing Gram-positive acid-fast bacterium. MAP can infect multiple animal species, but it causes clear clinical symptoms only in certain hosts such as ruminants and rabbits. In other animals, the infection is often asymptomatic [1,2]. Despite ongoing debate about its link to Crohn’s disease in humans, the presence of MAP in food has raised concerns within the food industry, prompting efforts to control Johne’s disease on farms [3].

The disease produces progressive enteritis in the form of diarrhea for a prolonged period, loss of weight, and decreased production essentially in small ruminants such as goats and sheep [4]. The disease is transmitted almost exclusively through the fecal–oral route, where young animals acquire the infection through contaminated milk, colostrum, or feeding material [5]. The bacterium is excreted in the feces and in milk for extended periods before the development of clinical disease and has been recovered from the semen and the reproductive tract material in some cases, suggesting more than a single mode of transmission [6,7]. MAP strains are broadly categorized into cattle (C-type) and sheep (S-type) based on host origin and into subtypes I and II by gene sequencing [4,8]. The disease progresses through four phases: silent infection, subclinical shedding, clinical disease, and progressive emaciation. Identification of MAP, particularly in the early stages, is still a problem [6]. While serological tests such as ELISA are commonly used for herd screening at the level, false negatives in the subclinical cases may be caused by the presence of low antibody titers [8]. Conversely, polymerase chain reaction (PCR) approaches achieves more sensitivity through the detection of bacterial DNA from direct clinical samples and are, therefore, particularly important in early-stage identification [9].

In regions like Saudi Arabia, there is little molecular and pathological data on infection by MAP in small ruminants. The gross pathology in Johne’s disease includes thickened intestinal walls, mucosa that is corrugated in type, and swollen mesenteric lymph nodes. Microscopical findings usually include involvement of the epithelioid macrophage, lymphocytes, and multinuclear giant cells. Granulomatous disease in the liver may show lepromatous as well as tuberculoid types [4,10]. In Saudi Arabia, very little molecular and pathological data exist on MAP infection in small ruminants, which hinders understanding of its prevalence and epidemiology. Correct diagnosis can comprise the integration of many approaches. Occasionally, animals were negative by ELISA but positive by real-time PCR, suggesting subclinical infection undetectable by antibody-based tests. Such findings illustrate the utility in the integration of molecular approaches within diagnostic workups, especially where early infection or latent infection is suspected [4,11,12]. To more accurately identify the strains, molecular genotyping can be conducted with the DMC gene in order to differentiate C-type and S-type strains and the IS900 gene in determining either subtype I or II. These two-markers method provides more in-depth analysis into the epidemiology and genetic variability of the MAP strains in circulation among the small ruminants [13]. This study analyzes Johne’s disease in Saudi sheep and goats through the integration of gross pathology, histopathology, serology (Ab ELISA), molecular diagnosis (real-time PCR), and strain genotyping. This approach is intended to raise the level of accuracy in disease diagnosis and produce new data on the existence of the disease and it’s molecular profile within the region.

## 2. Materials and Methods

### 2.1. Clinical Examination and Sampling

#### 2.1.1. Animals

**Figure 1:**
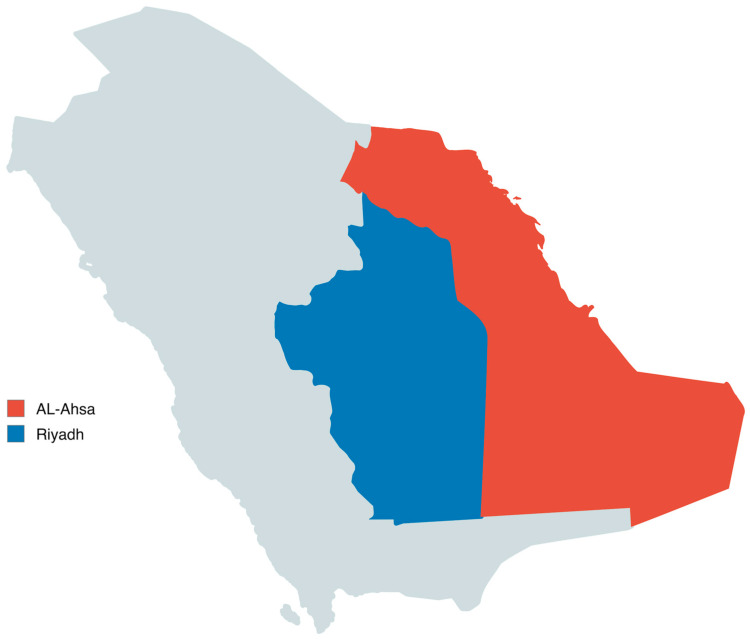
Map of Saudi Arabia showing the study areas in Riyadh (Central Region) and Al-Ahsa (Eastern Region), where small-ruminant samples were collected between 2021 and 2024.

A total of 388 locally raised small ruminants were included in this study (237 goats and 151 sheep). Animals aged 3–5 years were sampled between 2021 and 2024 from two major small-ruminant-rearing regions of Saudi Arabia: Riyadh (Central Region; 24.7136° N, 46.6753° E) and Al-Ahsa (Eastern Region; 25.3833° N, 49.5861° E) as shown in Figure 1 (Table 1). The animals were obtained from private herds presented to veterinary clinics and represented typical small-ruminant production systems in the studied regions, although detailed herd management and breeding data were not systematically recorded. Animals were recruited from herds with a documented history of *Mycobacterium avium* subsp. paratuberculosis (MAP) infection and included suspected clinical cases as well as apparently healthy in-contact animals. Blood (for serum) and fecal samples were collected from all animals, and all specimens were transported to the laboratory under chilled conditions. For pathological confirmation, necropsy was performed on a subset of 20 animals: 10 clinically progressed suspected cases and 10 apparently healthy in-contact animals from MAP-affected herds. The in-contact animals were included to assess possible subclinical infection and early lesions. Tissue specimens from the small and large intestine and mesenteric lymph nodes were collected and fixed in 10% neutral buffered formalin for histopathological examination. Ziehl–Neelsen (ZN) staining was performed on tissue impression smears from the necropsied subset as a supportive adjunct method to demonstrate acid-fast bacilli. Genotyping was performed only on tissue samples from six necropsied cases selected for molecular characterization. All procedures were approved by the King Faisal University Research Ethics Committee (Ref. No. KFU-REC-2022-NOV-ETHICS288). All efforts were made to minimize animal pain or suffering, and all sampling, necropsy, and laboratory procedures were conducted in accordance with national and international guidelines for the care and use of animals in research.

#### 2.1.2. ZN Staining

Ziehl–Neelsen (ZN) staining was used as a supportive adjunct method in necropsied cases by examining tissue impression smears from the intestine and mesenteric lymph nodes to demonstrate acid-fast bacilli.

#### 2.1.3. Ab Detection Screening ELISA

A total of 388 serum samples were screened for antibodies against *Mycobacterium avium* subsp. *paratuberculosis* (MAP) using a commercial indirect ELISA kit (Paratuberculosis Screening; IDEXX, Montpellier, France), following the manufacturer’s instructions. Sera were pre-incubated with *Mycobacterium phlei* to reduce non-specific reactivity. Optical density (OD) values were measured and expressed as sample-to-positive (S/P) ratios. Samples were classified as negative (≤0.45), suspect (0.45–0.55), or positive (≥0.55); suspect results were considered negative for statistical analyses.

#### 2.1.4. Histopathological Examination

Tissue specimens were fixed in 10% neutral buffered formalin, routinely processed, embedded in paraffin, sectioned, and stained with hematoxylin and eosin (H&E) for histopathological evaluation [14].

### 2.2. Molecular Detection and Genotyping of (MAP)

#### 2.2.1. DNA Extraction

Genomic DNA was extracted from fecal samples obtained from all animals (n = 388) using the GeneJET Genomic DNA Purification Kit (Thermo Scientific, Waltham, MA, USA) according to the manufacturer’s instructions, with minor modifications. For necropsied cases, DNA was additionally extracted from intestinal and mesenteric lymph node tissues for downstream genotyping/sequencing analyses.

#### 2.2.2. Real Time PCR Assay

Real-time PCR was performed using the VetMAX™ *M. paratuberculosis* 2.0 Kit (Applied Biosystems, Thermo Fisher Scientific, Waltham, MA, USA), which targets the IS900 insertion sequence. The 25 µL reaction mixture contained 12.5 µL master mix, 2 µL primer/probe mix, 5.5 µL nuclease-free water, and 5 µL DNA template. Amplification and fluorescence detection were performed on a LightCycler^®^ 480 real-time PCR system (Roche, Mannheim, Germany) under the following cycling conditions: 95 °C for 10 min, followed by 45 cycles of 95 °C for 15 s and 60 °C for 1 min. Results were interpreted according to the manufacturer’s instructions, with appropriate controls included in each run.

#### 2.2.3. Genotyping of MAP

Genotyping and sequencing was performed only on DNA extracted from intestinal and mesenteric lymph node tissues from six necropsied cases. PCR amplification was carried out using Invitrogen Platinum SuperFi II DNA Polymerase (Thermo Fisher Scientific, Waltham, MA, USA) according to the manufacturer’s instructions. Each 50 µL reaction contained 32.8 µL nuclease-free water, 5 µL 10× High-Fidelity PCR Buffer, 1 µL of 10 mM dNTP mix, 2 µL of 50 mM MgSO_4_, 0.2 µL of enzyme (5 U/µL), 2 µL of each primer, and 5 µL of DNA template. Three MAP-associated targets were amplified: IS900, DMC, and F57 [15,16,17]. The primers used are shown in Table 2.

PCR products were purified and sequenced bidirectionally using an Applied Biosystems 3500 automated DNA sequencer (ABI 3500, Foster City, CA, USA). Cycle sequencing was performed using the BigDye Terminator v3.1 kit (Applied Biosystems; Cat. No. 4336817), and sequencing was outsourced to Macrogen (Seoul, Republic of Korea). Raw chromatograms were trimmed and assembled to generate consensus nucleotide sequences, which were subsequently submitted to GenBank. Multiple sequence alignments were generated using ClustalW in BioEdit v7.7. Phylogenetic analysis was performed using the Maximum Likelihood method under the Tamura–Nei substitution model, and branch support was assessed by bootstrap analysis with 1000 replicates.

#### 2.2.4. Statistical Analysis

Statistical analyses were performed using SPSS software (version 20.0). Categorical variables were compared using the Chi-square test or Fisher’s exact test, as appropriate. Effect sizes were reported as odds ratios (ORs) with 95% confidence intervals (CIs). Multivariable logistic regression models were fitted to estimate adjusted odds ratios (aORs) for the association between species (goat vs. sheep) and MAP positivity by qPCR or ELISA, adjusting for region (Central vs. Eastern). Statistical significance was set at *p* < 0.05.

## 3. Results

A total of 388 animals were examined (151 sheep and 237 goats). IS900 real-time PCR detected MAP DNA in 135/388 animals (34.8%), while indirect ELISA detected MAP antibodies in 120/388 animals (30.9%). In sheep, 35/151 samples were qPCR-positive (23.1%) and 30/151 were ELISA-positive (19.8%). In goats, 100/237 samples were qPCR-positive (42.1%), and 90/237 were ELISA-positive (37.9%). Goats showed significantly higher qPCR positivity than sheep (42.1% vs. 23.1%; OR = 2.42, 95% CI: 1.53–3.82, *p* = 0.00013), and a higher ELISA seropositivity rate (37.9% vs. 19.8%; OR = 2.47, 95% CI: 1.53–3.98, *p* = 0.00017). After adjusting for region using multivariable logistic regression, goats remained significantly more likely to be qPCR-positive than sheep (aOR = 2.52, 95% CI: 1.58–4.00; *p* = 0.00009) and to be ELISA-positive (aOR = 2.58, 95% CI: 1.59–4.18; *p* = 0.00012). Overall, qPCR identified a higher proportion of MAP-positive animals than ELISA in both species. Ziehl–Neelsen (ZN) staining was performed on samples from the necropsied subset as supportive evidence; however, ZN results were not used for prevalence estimation due to limited sensitivity, particularly in subclinical/low-shedding animals. Among qPCR-positive animals (n = 135), cases were clinically staged as subclinical or clinically progressed based on the presence/absence of clinical signs.

### 3.1. Serological Detection of MAP Antibodies

Serum samples from 388 animals (237 goats and 151 sheep) were tested for antibodies against *Mycobacterium avium* subsp. paratuberculosis (MAP) using an indirect ELISA. Antibodies were detected in 120/388 animals (30.9%), including 90/237 goats (37.9%) and 30/151 sheep (19.8%) (Table 3).

### 3.2. Clinical and Pathological Findings

Pathological examination revealed distinct differences between subclinical and clinically advanced cases of Johne’s disease among sheep and goats.

**Figure 2:**
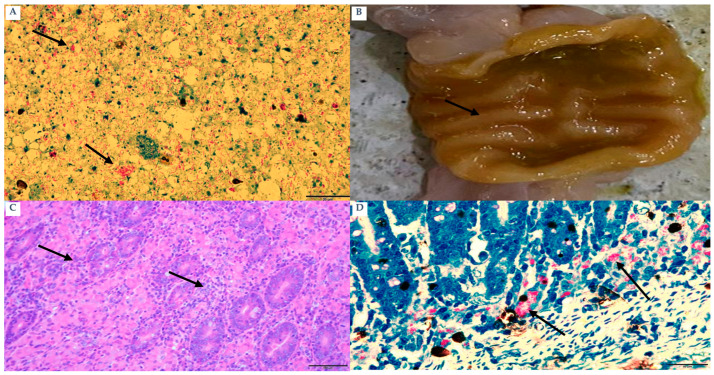
Subclinic animal: (**A**) Goat: Fecal smear stained with Ziehl–Neelsen stain shows clumps of acid-fast bacilli bacteria (arrows) bar 20 µm. (**B**) Goat: Ileum: The mucosal surface shows slightly transverse corrugation (arrow). (**C**) Goat: Ileum: tuberculoid reaction, multifocal aggregation of epithelioid macrophages intermingled with intestinal glands (arrows) (H&E) bar 100 µm. (**D**) Sheep: Ileum: Epithelioid and macrophages in the intestinal lamina propria shows clumps of acid-fast bacilli bacteria (arrows) (ZN stain) bar 60 µm.

Subclinical animals (n = 15; 10 goats and 5 sheep) showed no overt clinical signs and were classified as subclinical based on clinical examination. All subclinical animals were IS900 qPCR-positive (15/15). Ziehl–Neelsen (ZN) staining was positive in 10/10 subclinical animals. Gross and histopathological findings described below are based on the necropsied subset of subclinical cases (n = 10) (Figure 2A). ELISA findings were consistently negative, signifying the lack of a measurable humoral immune response. Intestinal lesions were predominantly modest and primarily confined to the ileum, with minor mucosal corrugation (Figure 2B). According to histopathology, the intestines had developed a multifocal granulomatous inflammation that was primarily composed of lymphocytes, with a little amount of macrophages and tuberculoid-type epithelioid cells present (Figure 2C). There were very few acid-fast bacilli found in macrophages and epithelioid cells (Figure 2D).

**Figure 3:**
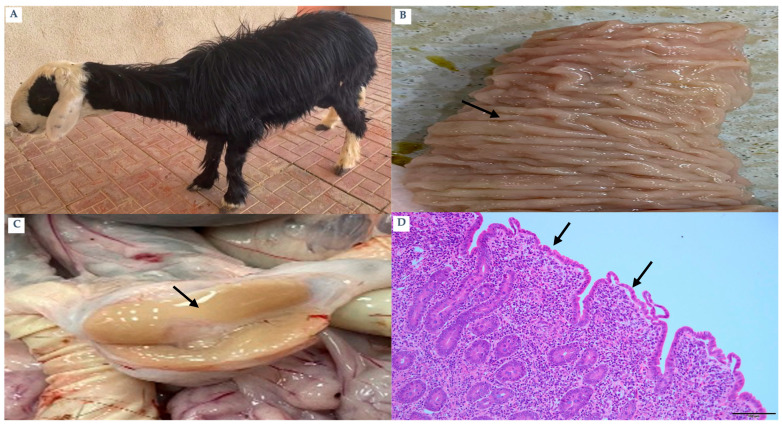
Clinic animal: (**A**) Sheep: 4-year-old. Lethargy, depressed and loss of appetite. (**B**) Goat: Ileum: The mucosal surface is diffusely thickened with rough undulated surface (arrows). (**C**) Sheep: Cross section of enlarged mesenteric lymph nodes showed yellow coloration (arrow). (**D**) Goat: Ileum: Blunt and short villi with expansion of lamina propria (arrows) (H&E) bar. 100 µm.

**Figure 4:**
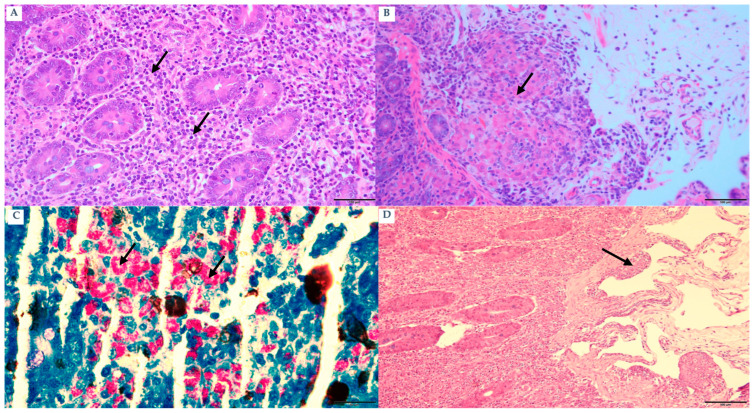
(**A**) Goat: Ileum: Higher magnification of the ileal lesion from the same goat shown in Figure 3D, illustrating diffuse granulomatous enteritis (H&E) bar 100 µm. (**B**) Goat: Ileum: Submucosal infiltration by epithloid cells (arrow), (H&E) bar 100 µm. (**C**) Sheep: Ileum: acid-fast bacilli in lamina propria (arrows) (ZN stain) bar 100 µm. (**D**) Sheep: Ileum: lymphangitis (arrow) and lymphangiectasia in submucosa (arrow) (H&E) bar 100 µm.

**Figure 5:**
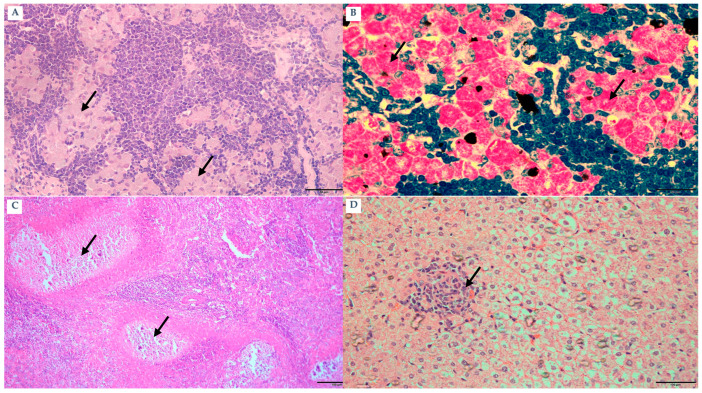
(**A**) Goat: Mesenteric lymph node: Clusters of epitheloid cells are intertwined with lymphoid follicles (arrows) (H&E) bar 100 µm. (**B**) Goat: Mesenteric lymph node: Clusters of epitheloid cells with acid-fast bacilli (arrows) (ZN stain) bar 100 µm. (**C**) Sheep: Mesenteric lymph node: multiple focal areas of massive caseous necrosis (arrows) (H&E) bar 100 µm. (**D**) Sheep: Liver: microgranuloma (arrow) (H&E) bar 100 µm.

Clinically progressed animals (n = 120; 90 goats and 30 sheep) exhibited hallmark manifestations of Johne’s disease, including progressive emaciation and poor body condition. Based on clinical examination, these animals were classified as clinically progressed; all clinically progressed animals were IS900 qPCR-positive (120/120) and ELISA-positive (120/120). ZN staining in the necropsied subset confirmed abundant acid-fast bacilli in clinically progressed cases. Detailed gross and histopathological findings described below are based on the necropsied subset of clinically progressed cases (n = 10) (Figure 3A). Inter-mandibular edema, commonly known as “bottle jaw,” was observed in multiple goats. All cases displayed affirmative results for both qPCR and ZN staining, whereas ELISA data indicated a high positivity rate. Gross lesions displayed notable thickening and a cord-like structure of the ileum, together with significant transverse mucosal corrugations (Figure 3B). The mesenteric and ileocecal lymph nodes demonstrated considerable hypertrophy (Figure 3C). Microscopically, diffuse granulomatous enteritis (lepromatous-type) was observed, characterized by blunted villi (Figure 3D) and significant infiltration of macrophages (Figure 4A) and epithelioid cells into the submucosa (Figure 4B). A significant quantity of acid-fast bacilli was detected within these cells (Figure 4C). Submucosal lymphangitis and lymphangiectasia were common (Figure 4D). Lymph nodes displayed considerable infiltration of epithelioid cells (Figure 5A), ZN-positive bacilli (Figure 5B), and caseous necrosis surrounded by macrophages, epithelioid cells, and multinucleated giant cells (Figure 5C). Some liver sections revealed foci of macrophages and lymphocytes (Figure 5D).

### 3.3. Molecular Identification of MAP Targeting IS900 Gene

IS900 real-time PCR detected *Mycobacterium avium* subsp. *paratuberculosis* (MAP) DNA in 135/388 animals, including subclinical cases (n = 15; 10 goats and 5 sheep) and clinically progressed cases (n = 120; 90 goats and 30 sheep). Cycle threshold (Ct) values among qPCR-positive samples ranged from 21 to 36, indicating variation in MAP DNA burden across samples. Positive and negative controls performed as expected in all runs, supporting the reliability of the assay. Overall, these results confirm the utility of IS900 qPCR for detecting MAP infection across a broad spectrum of clinical presentations, including clinically normal (subclinical) animals.

### 3.4. Genotyping and Subtyping of MAP Isolates Using DMC-PCR and IS900 Sequencing

Genotyping/subtyping was performed on tissue-derived DNA from six necropsied cases. DMC-PCR amplicon sizes were consistent with Type C (310 bp) in two cases (one goat, case 1; and one sheep, case 2) and Type S (162 bp) in four cases (two goats, cases 3 and 6; and two sheep, cases 4 and 5). IS900 sequence analysis identified a single nucleotide polymorphism (SNP) at nucleotide position 216, enabling classification relative to reference strains AE016958.1 (Type C/II; K-10) and CP033909.1 (Type S/I; MAPK).

**Figure 6:**
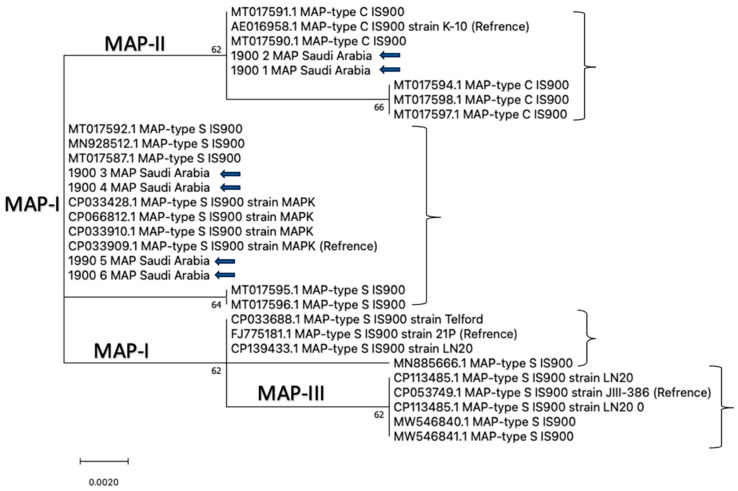
Maximum Likelihood phylogenetic tree based on partial IS900 sequences, showing the MAP-I, MAP-II, and MAP-III clades; the six study isolates cluster within MAP-I (Type S/I) and MAP-II (Type C/II). Bootstrap support values were estimated using 1000 replicates.

The purified IS900 PCR products were sequenced, and the resulting sequences were aligned with reference MAP strains retrieved from GenBank. Phylogenetic analysis was conducted in MEGA X using the Maximum Likelihood method under the Tamura–Nei substitution model, with branch support assessed using 1000 bootstrap replicates. The IS900 sequences generated in this study were deposited in GenBank under the following accession numbers: IS900-1 (goat; PX121622), IS900-2 (sheep; PX121621), IS900-3 (goat; PX121623), IS900-4 (sheep; PX121620), IS900-5 (sheep; PX121619), and IS900-6 (goat; PX115319). Consistent with DMC typing and the SNP at position 216, IS900-1 and IS900-2 (SNP = A) grouped with the Type C/II cluster, whereas IS900-3 to IS900-6 (SNP = G) grouped with the Type S/I cluster (Table 4; Figure 6). These findings indicate the concurrent circulation of distinct MAP types/subtypes among sheep and goats in the study regions.

## 4. Discussion

The present study demonstrated that Johne’s disease is prevalent among sheep and goats in Saudi Arabia and highlights the value of integrating clinical evaluation with molecular, serological, and pathological assessments. Animals were categorized into subclinical and clinically progressed stages based on clinical examination, while qPCR, ELISA, and supportive microscopy provided complementary evidence across the disease spectrum. In this study, qPCR positivity was higher in goats (42.1%) than in sheep (23.1%), indicating a higher MAP detection rate in goats; accordingly, goats showed significantly higher odds of qPCR positivity than sheep (OR = 2.42, 95% CI: 1.53–3.82). Although Ziehl–Neelsen (ZN) staining can provide supportive microscopic evidence of acid-fast bacilli, it is known to have limited sensitivity, particularly in subclinical or low-shedding cases; therefore, ZN results were not used for prevalence estimation in this study. Overall, IS900 real-time PCR proved more sensitive than ZN staining and provided a MAP-specific diagnostic approach for detecting infection, particularly in subclinical animals with low or intermittent shedding. Similar observations have been reported previously [7,18]. In addition to molecular detection and supportive staining, indirect ELISA is widely applied as a herd-level serological tool to support the diagnosis of paratuberculosis. In the present study, ELISA detected MAP antibodies in 120/388 animals (30.9%), with a higher seroprevalence in goats (90/237; 37.9%) than in sheep (30/151; 19.8%); accordingly, goats showed higher odds of ELISA seropositivity than sheep (OR = 2.47, 95% CI: 1.53–3.98). Notably, ELISA results were negative in all subclinical animals (0/15) and positive in all clinically progressed cases (120/120), which is consistent with the concept that humoral immune responses become more readily detectable in advanced stages of MAP infection. Similar findings have been reported previously [18]. Therefore, ELISA can be useful for identifying animals with more advanced disease when interpreted alongside clinical assessment and pathological findings, whereas qPCR remains valuable for detecting infection across both subclinical and clinical stages. Moreover, goats showed significantly higher odds of MAP positivity than sheep by both qPCR and ELISA, and this association remained significant after adjusting for region in multivariable logistic regression. Collectively, these findings support a diagnostic hierarchy in which qPCR is optimal for early/subclinical detection, ELISA is more informative in advanced stages, and ZN staining mainly supports confirmation of high-burden cases. Clinically, goats and sheep showed broadly similar manifestations; however, goats tended to exhibit more pronounced disease progression, in agreement with previous findings [19]. A close relationship has been described between clinical presentation, pathological changes, and the serological response in paratuberculosis, which is consistent with the stage-dependent diagnostic patterns observed in the present study [20]. In the early clinical stage, animals may exhibit depression, reduced appetite, hair loss, and poor body condition, which may correspond to mild-to-moderate intestinal lesions, including focal granulomatous enteritis [1]. In advanced stages, animals often develop severe emaciation, progressive weight loss, dehydration, and “bottle jaw”, particularly in goats, which is typically associated with hypoproteinemia secondary to chronic intestinal disease. These clinical manifestations are consistent with more severe intestinal pathology such as diffuse granulomatous enteritis, lymphangitis, and lymphangiectasia [21]. Grossly, intestinal thickening, mucosal corrugation, serous atrophy of fat, and enlargement of mesenteric and ileocecal lymph nodes were more evident in goats, whereas sheep showed variable intestinal thickening with prominent involvement near the ileocecal junction [1,22]. Histopathologically, lesions were generally more severe in goats than in sheep and were broadly consistent with two major patterns: tuberculoid and lepromatous forms. The tuberculoid form is characterized by limited numbers of acid-fast organisms with prominent cellular immune responses, often observed in early infection and subclinical disease [23]. In contrast, the lepromatous form is marked by abundant acid-fast organisms within macrophages and epithelioid cells and is typically associated with advanced clinical disease and a more pronounced humoral immune response [24,25]. This spectrum of pathology supports the observed diagnostic patterns, in which subclinical infection was more readily detected by qPCR, while clinically progressed cases were consistently ELISA-positive. Molecular confirmation and strain typing further demonstrated the genetic heterogeneity of MAP circulating in the investigated regions. IS900-based qPCR was used for molecular detection, while additional targets (F57 and DMC) were applied to tissue-derived DNA from selected necropsied cases to confirm MAP specificity and support typing/subtyping [4,18,26]. Furthermore, DMC-based typing and IS900 sequence analysis (SNP at position 216) demonstrated the concurrent circulation of both cattle-type (C/II) and sheep-type (S/I) MAP strains among the investigated animals, consistent with previous reports that MAP types are not strictly host-restricted [27]. Phylogenetic analysis of the IS900 sequences generated in this study showed that isolates clustered into MAP-I (S-type) and MAP-II (C-type) groups, consistent with DMC typing and SNP-based classification. Specifically, two isolates clustered with the Type C/II reference strain AE016958.1, while four isolates clustered within the Type S/I group alongside CP033909.1 and related reference strains, highlighting diverse evolutionary lineages within the regional MAP population. Similar subtype-defining SNP patterns at position 216 have been reported elsewhere [4,28].

## 5. Conclusions

This study provides a comprehensive integrated pathological, molecular, and genotypic characterization of *Mycobacterium avium* subsp. *paratuberculosis* (MAP) infection in sheep and goats in Saudi Arabia. The combined application of histopathology, serological screening, and multi-marker molecular detection revealed clear differences between subclinical and clinically progressed infections, highlighting the superior sensitivity of IS900 real-time PCR for early detection. In this study, qPCR detected MAP DNA in 34.8% of animals, with higher positivity in goats than in sheep, whereas ELISA more effectively identified clinically progressed cases, consistent with stage-dependent humoral responses. This difference remained significant after adjusting for region (qPCR: aOR = 2.52; ELISA: aOR = 2.58). Genotyping based on DMC typing and IS900/F57 targets confirmed the concurrent circulation of both Type C and Type S MAP strains, indicating genetic heterogeneity within the regional MAP population. Collectively, these findings improve current understanding of MAP epidemiology in small ruminants and support the implementation of integrated molecular–pathological diagnostic strategies to strengthen surveillance and control programs in Saudi Arabia.

## Figures and Tables

**Table 1:** Data of samples collected during this study including species, location and number.

Herd	Sheep		Goats	
	Number	Location	Number	Location
1	15	Eastern region	38	Eastern region
2	10	Eastern region	45	Eastern region
3	23	Eastern region	20	Eastern region
4	20	Central region	63	Central region
5	30	Central region	28	Central region
6	40	Central region	30	Central region
7	4	Central region	3	Central region
8	9	Central region	10	Eastern region
Total	151	Total	237	

**Table 2:** Primers used in this study for partial amplification of IS900, F57 and DMC1 genes of MAP.

Primer’s Name	Sequence	Target Gene	Expected Product (bp)	Annealing Temp	Reference
IS900F IS900R	CCTTTCTTGAAGGGTGTTCG CCACCAGATCGGAACGTC	IS900	548 bp	59 °C	[15]
F57 F F57 R	CCCGATAGCTTTCCTCTCCT GATCTCAGACAGTGGCAGGTG	F57	600 bp	57 °C	[16]
DMC-529 F DMC1-531 F DMC1-533 R	GCTGTTGGCTGCGTCATGAAG TCTTATCGGACTTCTTCT GGC CGGATTGACCTGCGTTTCAC	DMC1	310 (C-Type) 162 (S-Type)	60 °C	[17]

**Table 3:** Distribution of sampled animals by region and diagnostic positivity (qPCR and ELISA).

Species	Region	n	qPCR+ n (%)	ELISA+ n (%)
Goats	Al-Ahsa (Eastern)	89	46 (51.7)	41 (46.1)
Goats	Riyadh (Central)	148	54 (36.5)	49 (33.1)
Sheep	Al-Ahsa (Eastern)	72	15 (20.8)	14 (19.4)
Sheep	Riyadh (Central)	79	20 (25.3)	16 (20.3)
Total		388	135 (34.8)	120 (30.9)

**Table 4:** Molecular characterization of tissue-derived DNA from six necropsied cases selected for DMC typing, IS900/F57 PCR confirmation, and partial IS900 sequencing (n = 6).

No. ID	Host	ZN	ELISA	rtPCR	CT	IS900	F57	SNPs (216)	DMC Type	Subtype
IS900 1/PX121622	Goat	+VE	+VE	+VE	22.1	+VE	+VE	A	C	II
IS900 2/PX121621	Sheep	+VE	+VE	+VE	25.6	+VE	+VE	A	C	II
IS900 3/PX121623	Goat	+VE	+VE	+VE	25.45	+VE	+VE	G	S	I
IS900 4/PX121620	Sheep	+VE	+VE	+VE	24.8	+VE	+VE	G	S	I
IS900 5/PX121619	Sheep	+VE	+VE	+VE	25.08	+VE	+VE	G	S	I
IS900 6/PX115319	Goat	+VE	+VE	+VE	23.5	+VE	+VE	G	S	I

## Data Availability

The data supporting the findings of this study are available from the corresponding author upon reasonable request. The IS900 sequences generated in this study have been deposited in GenBank under accession numbers PX115319 to PX121623.
